# Evolution of Systemic Treatment Uptake and Survival in Advanced Non-Small Cell Lung Cancer

**DOI:** 10.3390/curroncol28010008

**Published:** 2020-12-04

**Authors:** Sophie Stock-Martineau, Katie Laurie, Mathieu McKinnon, Tinghua Zhang, Paul Wheatley-Price

**Affiliations:** 1 Department of Medicine, University of Ottawa, Ottawa, ON K1H 8L6, Canada; sstock@toh.ca; 2 The Ottawa Hospital Research Institute, Ottawa, ON K1Y 4E9, Canada; katielaurie18@gmail.com (K.L.); mmcki097@uottawa.ca (M.M.); tizhang@ohri.ca (T.Z.)

**Keywords:** non-small-cell lung cancer, real-world data, chemotherapy, epidermal growth factor receptor, anaplastic lymphoma kinase, immune checkpoint inhibitor

## Abstract

Background: Non-small cell lung cancer (NSCLC) commonly presents at advanced stage. We previously reported systemic treatment uptake in stage IV NSCLC climbing from 55% (2009–2012) to 62% (2015–2017). Since then, first-line immunotherapy and 2nd/3rd generation tyrosine kinase inhibitors (TKIs) have emerged as standards of care. We explored whether treatment rates continued to rise and studied outcomes. Methods: We reviewed all cases of de novo stage IIIB/IIIC/IV NSCLC seen in out-patient medical oncology consultation at our institution between 2009–2012 (cohort A), 2015–2017 (cohort B), and June–December 2018 (cohort C). We compared rates of systemic treatment, molecular testing, targeted therapy, and immune checkpoint inhibitor (ICI) use. We compared survival in the overall, treated/untreated, younger and elderly population in cohort A vs. cohort B + C (=cohort D). Results: Cohorts A, B, and C included 528, 463, and 93 patients, respectively. Overall, 66% received any systemic therapy in cohort C, compared to 62% in cohort B and 55% in cohort A. Across three time periods, first-line chemotherapy rates fell (93, 76, 46%) while rates of first-line targeted therapy (5, 16, 15%) and ICI (0, 2, 36%) rose. Among molecular subtypes, first-line targeted treatment in EGFR-positive patients (63, 94, 100%) and anaplastic lymphoma kinase (ALK)-positive patients (0, 91, 100%) rose. Survival improved in all subgroups in cohort D vs. cohort A, except for patients ≥ 70 years and the untreated population. Conclusions: Systemic treatment rose across three time periods, reflecting the introduction of rapid diagnostic pathways, reflex molecular testing, ICI, and targeted therapies. Survival outcomes of advanced NSCLC patients have significantly improved.

## 1. Introduction

Lung cancer remains an important disease burden worldwide. According to the World Health Organization (WHO), there were 2.09 million cases of lung cancer in 2018, internationally [1]. This represents the 6th most common cause of death worldwide, and the leading cause of cancer death. Likewise, in Canada, lung malignancy is the leading cause of cancer-related deaths. In 2020, it is estimated that one-quarter of cancer mortality will be attributable to lung cancer [2]. Unfortunately, the overall survival (OS) rate remains poor with only 19.2% of patients still being alive at 5 years [3]. Brule et al. published a review of advanced non-small cell lung carcinoma (NSCLC) cases seen at the Ottawa Hospital Cancer Centre between 2009–2012. Of 528 patients, only 55% received any palliative systemic therapy [4]. When we compared this cohort to a more recent Ottawa Cancer Center database including metastatic NSCLC seen between 2015–2017, the proportion of patients receiving palliative systemic therapy had increased to 62% [5].

In the last decade, many new therapies were approved as first-line treatment for stage IV NSCLC. In 2016, first-line immune checkpoint inhibitors (ICI) such as anti-programmed death 1 (PD-1) monoclonal antibody pembrolizumab were reported as a new standard of care for treatment-naïve NSCLC with programmed death-ligand 1 (PDL1) expression of 50% and more, however Ontario public funding of this only commenced in January 2018 [6]. The combination of chemotherapy and ICI was also recently approved as first-line therapy for advanced NSCLC without driver mutation and regardless of PDL1 expression [7,8]. Second and third generation tyrosine kinase inhibitors (TKI) have emerged in the front-line setting for NSCLC with driver mutations. In 2018, osimertinib showed superior progression-free survival (PFS) and in 2020, improved median OS, compared to first-generation TKIs gefitinib or erlotinib in epidermal growth factor receptor (EGFR) mutation-positive NSCLC [9,10]. Alectinib, a second generation TKI, has shown considerable PFS benefit compared to first-generation TKI crizotinib in anaplastic lymphoma kinase (ALK)-positive disease [11]. The updated cohort previously mentioned from 2015–2017 had just completed data collection prior to the uptake of first-line immunotherapy. Given the recent availability of these effective drugs, we explored whether treatment uptake rose further and whether survival outcomes according to treatment type and age improved over time.

## 2. Experimental Section

### 2.1. Patient Data

With ethics approval (Research Ethics Board; 638; 22 May 2018, no informed consents were signed as this was a retrospective chart review), we reviewed cases of de novo stage IIIB/IIIC/IV NSCLC seen in out-patient medical oncology consultation at the Ottawa Hospital Cancer Center between 2009–2012, 2015–2017, and June-December 2018. The Ottawa Hospital Cancer Centre is a university-affiliated academic tertiary referral centre and is the sole provider of lung cancer services to a population of nearly 1.5 million in Eastern Ontario. Patient demographics, disease characteristics, molecular analysis, treatment types, and survival outcomes according to age and treatment were gathered from hospital records. Patients included were assessed in consultation as an outpatient for a new diagnosis of confirmed stage IIIB/IIIC with palliative intent or stage IV NSCLC. Exclusion criteria were patients first seen as an in-patient and patients who had recurrent lung cancer after previous curative-intent therapy. Methods have also been previously described [4].

### 2.2. Statistical Methods

We performed a retrospective descriptive analysis comparing patients during three different time periods: cohort A: 2009–2012, B: 2015–2017, and C: June to December 2018. For further analysis we combined cohorts B and C to create cohort D, leading to an earlier cohort A (2009–2012) and a later cohort D (2015–2018). We reported age at diagnosis as a median and a range. Other baseline characteristics were reported with number of patients and rates (percentage). The Kaplan-Meier method was used to compare overall survivals between two time periods (cohort A vs. D) according to different treatment groups and ages. P-values were determined by using the log-rank test. All analyses were conducted using SAS version 9.4 (SAS Institute Inc, Cary, NC, USA).

## 3. Results

### 3.1. Baseline Patient Characteristics of Three Time Periods

Baseline patient characteristics are shown in Table 1. Cohorts A, B, and C included 528, 463, and 93 patients, respectively. The median ages were 68, 69, and 69. The male to female ratio remained similar (55/45, 55/45, 56/44). Adenocarcinoma frequency increased (58, 74, 75%) whereas squamous cell rates decreased (22, 18, 17%) over time. Testing for PDL1 in all NSCLC (0, 6, 97%), EGFR (21, 83, 95%), and ALK (7, 89, 96%) testing in non-squamous histology also rose across time periods.

### 3.2. Systemic Therapy

**Figure 1:**
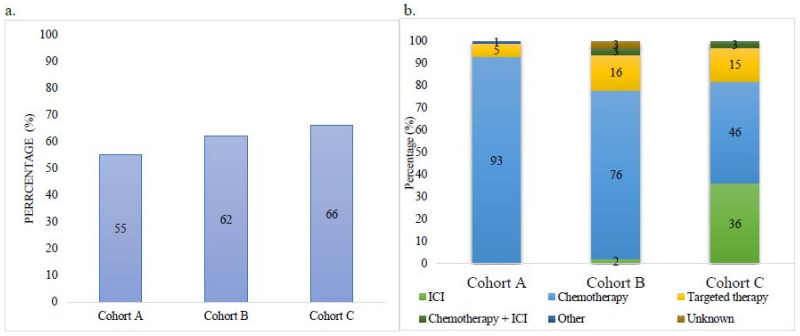
(**a**) Rate of first-line palliative systemic treatment in each cohort in patients with stage IV non-small cell lung carcinoma (NSCLC); (**b**) rate of first line therapies for each cohort. Percentages may not total 100 because of rounding.

Systemic palliative treatment uptakes are shown in Figure 1a. Overall, 66% received any systemic therapy in cohort C, compared to 62% in cohort B, and 55% in cohort A. Across three time periods, among those receiving at least one line of treatment, the rate of cytotoxic chemotherapy fell (93, 76, 46%) while rates of first-line targeted therapy (5, 16, 15%) and ICI (0, 2, 36%) rose (Table 2) (Figure 1b).

Among molecular subtypes, there were rises in first-line targeted treatment in EGFR-positive patients (63, 94, 100%) and ALK-positive patients (0, 91, 100%) (Table 3).

Second-line targeted therapy is not reported in this manuscript due to lack of follow-up and a high proportion of patients still continuing on first-line therapy (Table 3).

### 3.3. Survival Outcomes

**Figure 2:**
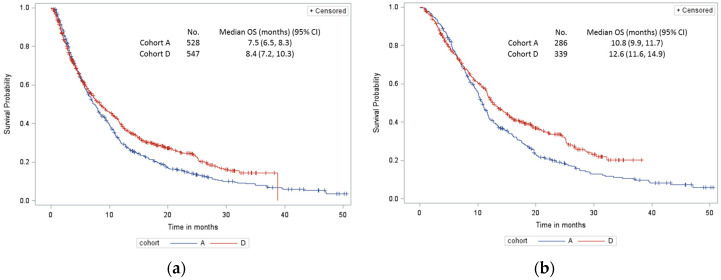

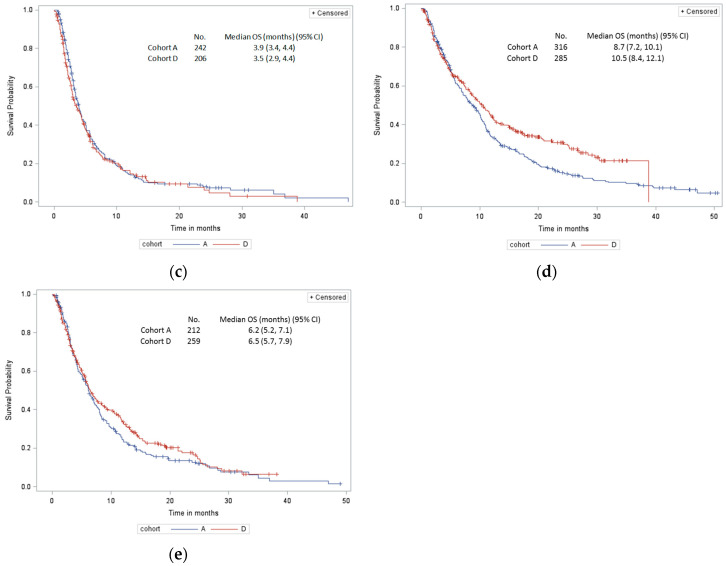
Overall survival (OS) of all patients with stage IV non-small cell lung carcinoma (NSCLC) in cohort A vs. D: (**a**) all patients treated and untreated; (**b**) treated patients only; (**c**) untreated patients only; (**d**) patients < 70 years; (**e**) patients ≥ 70 years. CI, confidence interval.

For comparative OS analysis between different time periods, patients in cohort B were combined to those in cohort C, which resulted in cohort D. Median OS was longer in cohort D compared to cohort A (8.4 vs. 7.5 months, *p* < 0.01) (Figure 2a). Among patients who received any systemic therapy, OS was longer in cohort D than cohort A patients (12.6 vs. 10.8 months, *p* <0.01) (Figure 2b). Survival was poor and not statistically significantly different among untreated patients in cohort A vs. D (3.9 vs. 3.5 months, *p* < 0.35) (Figure 2c). When analyzing by age, in treated/untreated younger patients (less than 70 years), median OS was also longer in cohort D compared to cohort A (10.5 vs. 8.7 months, *p* < 0.01) (Figure 2d). There was no statistically significant difference in median OS for treated/untreated patients 70 years or above between cohorts A vs. D (6.2 vs. 6.5 months, *p* <0.24) (Figure 2e).

## 4. Discussion

Before the approval of either ICIs or TKIs, palliative platinum-based chemotherapy remained the primary standard first-line therapy for advanced NSCLC, with single-agent chemotherapy occasionally prescribed in elderly patients or those with borderline functional status. Systemic chemotherapy provides symptom palliation and extends life. In the earliest platinum trials, platinum-based chemotherapy reduced risk of death by 27% and increased survival at 1 year by 10% compared to best supportive care alone [12]. Those outcomes were further improved with the widespread adoption of modern platinum doublets. In the last decade, with the introduction of the biomarker, PDL1, and driver mutation testing, new frontline non-chemotherapy agents have emerged.

Canadian practice guidelines on standardized molecular testing in advanced NSCLC were published in 2018. Melosky et al. recommend systematic testing of PDL1, EGFR, ALK, UR2 sarcoma virus oncogene homolog 1 (ROS1), and BRAF in non-squamous histologies, and PDL1, and EGFR (non-smokers only) in squamous NSCLC [13]. In 2018, the European Society for Medical Oncology (ESMO) published updated clinical practice guidelines for biomarker/molecular testing and treatment algorithms for advanced NSCLC. They recommend systematic biomarker/molecular testing including PDL1, EGFR, ALK, ROS1, and BRAF for all non-squamous cell carcinomas. They propose a treatment algorithm for NSCLC without a driver mutation in the frontline setting. Pembrolizumab is recommended if PDL1 is 50% or higher. Other options for patients with performance status 0–2 are platinum-based chemotherapy/ICI or platinum-based chemotherapy alone. For cancers with a driver mutation, frontline TKI is favored to chemotherapy [14].

Our study reflects a shift in frontline treatment for advanced NSCLC. In this single-institution academic centre review, with the recent introduction of reflex molecular testing, ICI and targeted therapies, overall palliative systemic treatment uptake has risen across three time periods. Oncologists treating lung cancer were hopeful that frontline systemic treatment would move away from intravenous infusions and that oral therapies would predominate. However, as our study shows, first-line ICI rates have risen significantly. Furthermore, Brule et al. showed that 51% of patients in our cohort A had received second-line treatment [4]. With longer follow-up, it will be interesting to see if second-line therapy uptakes rise further in our patients with advanced NSCLC, including those with driver mutations.

As mentioned above, our study confirms increasing rates of frontline systemic treatment. These findings led to improvement in survival over time in the overall, treated, and younger population (less than 70 years old). Our data are consistent with a Japanese single-center retrospective study. With a total of 1547 patients with stage IV NSCLC, it reported improvement in OS throughout five time periods. Our study did confirm an improved survival in a Caucasian population with advanced NSCLC. Interestingly, Takano et al. reported higher and a similar trend of increasing rates of never smokers with NSCLC (33% in 2015–2017, 17% in 1990–1995 vs. our cohorts 10% in cohort C, 13% in cohort B, and 7% in cohort A). As expected, the Japanese population had higher rates of EGFR and ALK-positive lung cancer (31 and 6%, respectively in 2015–2017) [15].

In this study, survival amongst untreated patients in our cohort remained poor and did not improve, whereas the treated population showed prolonged OS over time. We can thus conclude that utilization of better treatments such as ICIs and targeted therapies translate into improved survival outcomes in the treated vs. untreated population. Furthermore, routine use of PET scans in the early staging of NSCLC has shown upstaging due to extrathoracic metastasis in 15% of patients [16]. Stage migration with PET scans and detection of early metastatic or oligometastatic disease might also contribute to better survival trends seen in the treated population over time. Unfortunately, in our study, median OS in patients 70 years and above did not improve when comparing two distinct time periods. Elderly patients are often sicker with more comorbidities and poorer performance status [17]. They often experience increased hematological toxicity related to chemotherapy [18]. In addition, polypharmacy in the elderly also makes interference with chemotherapy metabolism more likely [19]. Further studies will be needed to investigate this group, as theoretically the newer therapies should be more tolerable in an elderly, frail, or comorbid subgroup, so with more time the survival advantages hopefully would also be seen in the elderly.

This study does have some limitations. It is a retrospective analysis and represents a single-center experience. Cohort C consists of a small number of patients and is unbalanced when compared to Cohorts A and B. Therefore, the degree of overall survival improvement over time might be underestimated. There is a pan-Canadian lung cancer observational study (PALEOS) now being developed, that in time will have much greater power to look at these questions.

At our center, all newly diagnosed stage IV non-squamous NSCLC now undergo molecular studies including EGFR, ALK, ROS1, BRAF, and KRAS. Biomarker PDL1 is tested in all NSCLCs. Next generation sequencing is becoming more common, and with the emergence of more druggable targets (BRAF V600E, KRAS G12C, RET fusion, c-MET skipping mutations, NTRK fusions, etc.), further improvements in treatment rates and survival can be anticipated. The KRAS G12C mutation is of particular interest as it could represent a subgroup of a similar size to EGFR mutations (10–15%), and the KRAS G12C inhibitor, AMG 510, has shown promising activity in a phase I trial in NSCLC [20]. For patients without any driver mutation and PDL1 less than 50%, first-line combination pembrolizumab and platinum-based chemotherapy will be a further advance almost ready to be rolled out in the clinic, although this may not increase overall treatment rates, as those patients will still need to have sufficient functional status to tolerate the platinum component.

## 5. Conclusions

In summary, we believe systemic treatment uptake and survival outcomes should continue to improve with widespread testing of driver mutations, use of their corresponding targeted therapies, and emergence of novel therapies. While the 5-year survival rates in lung cancer remain, at 19%, unacceptably low, there is progress and in coming years we would anticipate more substantial incremental gains [3].

## Figures and Tables

**Table 1:** Baseline characteristics of patients in each cohort.

Characteristics	Cohort A	Cohort B	Cohort C
	*N* (%)	*N* (%)	*N* (%)
Number of Patients (total)	528	463	93
Age at Diagnosis Median Years and (Range)	68 (35–90)	69 (23–94)	69 (39–89)
Male/female	292 (55)/236 (45)	256 (55)/207 (45)	52 (56)/41 (44)
ECOG 0	46 (9)	41 (9)	9 (10)
ECOG 1	220 (42)	177 (38)	33 (35)
ECOG 2	111 (21)	101 (22)	16 (17)
ECOG 3	92 (17)	56 (12)	21 (23)
ECOG 4	19 (4)	14 (3)	3 (3)
Unknown	40 (8)	74 (16)	11 (12)
Current Smoker	228 (43)	196 (42)	43 (46)
Ex-Smoker	257 (49)	207 (45)	40 (43)
Never Smoker	37 (7)	58 (13)	9 (10)
Unknown	6 (1)	2 (0.4)	1 (1)
Adenocarcinoma	308 (58)	344 (74)	70 (75)
Squamous Cell	118 (22)	82 (18)	16 (17)
Large Cell	27 (5)	9 (2)	0
Other NSCLC/NOS	30 (6)/0	9 (2)/19 (4)	3 (3)/4 (4)
Unknown	45 (9)	0	0
Stage IIIB/IIIC/IV	35 (7)/0/493 (93)	36 (8)/0/427 (92)	5 (5)/1 (1)/83 (89)
Unknown	0	0	4 (4)
PDL1 tested *	N/A	26 (6)	90 (97)
Not Tested	N/A	434 (94)	2 (2)
Unknown	N/A	3 (0.7)	1 (1)
50% and Higher		10 (38)	37 (41)
1–49%		2 (8)	19 (21)
<1%	N/A	13 (50)	24 (27)
Insufficient Sample		0	10 (11)
Not Tested		1 (4)	0
Non-Squamous	365 (69)	381 (82)	77 (83)
EGFR Tested **	77 (21)	316 (83)	73 (95)
Negative	59 (77)	247 (78)	60 (82)
Positive	18 (23)	42 (13)	9 (12)
Inconclusive	0	27 (9)	4 (5)
Not Tested	288 (79)	65 (17)	4 (5)
L858R	N/A	18 (43)	3 (33)
Exon 19 Deletion	N/A	17 (40)	4 (44)
Other	N/A	7 (17)	2 (22)
Unstated ***	18 (100)	0	0
Non-Squamous	365 (69)	381 (82)	77 (83)
ALK tested **	24 (7)	338 (89)	74 (96)
Negative	20 (83)	313 (93)	71 (96)
Positive	4 (17)	12 (4)	2 (3)
Inconclusive	0	13 (4)	1 (1)
Not Tested	341 (93)	43 (11)	3 (4)

ECOG, Eastern Cooperative Oncology Group; PS, performance status; NSCLC, non-small cell lung carcinoma; NOS, not otherwise specified; PDL1, programmed death-ligand 1; EGFR, epidermal growth factor receptor; ALK, anaplastic lymphoma kinase; N/A, not applicable; * PDL1 testing was not yet available in cohort A; ** EGFR and ALK testing are reported in patients with non-squamous histology; *** Specific EGFR mutation testing was not available in cohort A. Percentages may not total 100 because of rounding.

**Table 2:** First-line palliative systemic treatment in each cohort.

Treatment as First Line	Cohort A *N* (%)	Cohort B *N* (%)	Cohort C *N* (%)
Number of Patients (Total)	528	463	93
No Treatment Received	237 (45)	176 (38)	31 (33)
Treatment Unknown	0	0	1 (1)
Received Treatment	291 (55)	287 (62)	61 (66)
ICI	0	5 (2)	22 (36)
Chemotherapy	272 (93)	219 (76)	28 (46)
Targeted Therapy	16 (5)	46 (16)	9 (15)
Chemotherapy + ICI	0	8 (3)	2 (3)
Other (Study Drug)	3 (1)	0	0
Unknown	0	9 (3)	0
Patients ≥70 Years Old	212 (40)	224 (48)	44 (47)
Received Treatment /No Treatment	78 (37)/134 (63)	114 (51)/110 (49)	23 (52)/20 (48) 1 treatment unknown
Patients <70 Years Old	316 (60)	239 (52)	49 (53)
Received Treatment /No Treatment	208 (66)/108 (34)	173 (72)/66 (28)	38 (78)/11 (22)

ICI, immune checkpoint inhibitor. Percentages may not total 100 because of rounding.

**Table 3:** First-line treatment in EGFR and ALK-positive patients in each cohort.

Treatment	Cohort A	Cohort B	Cohort C
	*N* (%)	*N* (%)	*N* (%)
Number of Patients EGFR-Positive	20 (24)	42 (13)	9 (12)
1st Line	16 (80)	38 (90)	7 (78)
Targeted Therapy	10 (63)	36 (94)	7 (100)
Chemotherapy	6 (38)	1 (3)	0
Unknown	0	1 (3)	0
Number of Patients ALK-Positive	4 (17)	12 (4)	2 (3)
1st Line	4 (100)	11 (92)	2 (100)
Targeted Therapy	0	10 (91)	2 (100)
Chemotherapy	4 (100)	1 (9)	0
Unknown	0	0	0

EGFR, epidermal growth factor receptor; ALK, anaplastic lymphoma kinase; ICI, immune checkpoint inhibitor.

## References

[1] World Health Organization (2018). Lung Cancer Statistics. https://www.who.int/news-room/fact-sheets/detail/cancer.

[2] Canadian Cancer Society (2020). Lung Cancer Statistics. https://www.cancer.ca/en/cancer-information/cancer-type/lung/statistics/?region=pe.

[3] Lung and Bronchus Cancer (2019). Surveillance, Epidemiology, and End Results Program. http://seer.cancer.gov/statfacts/html/lungb.html.

[4] Brule S.Y., Al-Baimani K., Jonker H., Zhang T., Nicholas G., Goss G., Laurie S.A., Wheatley-Price P. (2016). Palliative systemic therapy for advanced non-small cell lung cancer: Investigating disparities between patients who are treated versus those who are not. Lung Cancer.

[5] Wheatley-Price P., Laurie K., Zhang T., McKinnon M. (2019). Evolution of Systemic Therapy Uptake in NSCLC Over Time: The Impact of New Therapies [abstract P1.01-16]. J. Thorac. Oncol..

[6] Reck M., Rodríguez-Abreu D., Robinson A.G., Hui R., Csőszi T., Fülöp A., Gottfried M., Peled N., Tafreshi A., Cuffe S. (2016). Pembrolizumab versus Chemotherapy for PD-L1-Positive Non-Small-Cell Lung Cancer. N. Engl. J. Med..

[7] Gandhi L., Rodríguez-Abreu D., Gadgeel S., Esteban E., Felip E., De Angelis F., Domine M., Clingan P., Hochmair M.J., Powell S.F. (2018). Pembrolizumab plus Chemotherapy in Metastatic Non-Small-Cell Lung Cancer. N. Engl. J. Med..

[8] Paz-Ares L., Luft A., Vicente D., Tafreshi A., Gümüş M., Mazières J., Hermes B., Şenler F.C., Csőszi T., Fülöp A. (2018). Pembrolizumab plus Chemotherapy for Squamous Non-Small-Cell Lung Cancer. N. Engl. J. Med..

[9] Soria J.-C., Ohe Y., Vansteenkiste J., Reungwetwattana T., Chewaskulyong B., Lee K.H., Dechaphunkul A., Imamura F., Nogami N., Kurata T. (2018). Osimertinib in Untreated EGFR-Mutated Advanced Non-Small-Cell Lung Cancer. N. Engl. J. Med..

[10] Ramalingam S.S., Vansteenkiste J., Planchard D., Cho B.C., Gray J.E., Ohe Y., Zhou C., Reungwetwattana T., Cheng Y., Chewaskulyong B. (2020). Overall Survival with Osimertinib in Untreated, EGFR-Mutated Advanced NSCLC. N. Engl. J. Med..

[11] Hida T., Nokihara H., Kondo M., Kim Y.H., Azuma K., Seto T., Takiguchi Y., Nishio M., Yoshioka H., Imamura F. (2017). Alectinib versus crizotinib in patients with ALK-positive non-small-cell lung cancer (J-ALEX): An open-label, randomised phase 3 trial. Lancet.

[12] Non-small Cell Lung Cancer Collaborative Group (1995). Chemotherapy in non-small cell lung cancer: A meta-analysis using updated data on individual patients from 52 randomised clinical trials. BMJ.

[13] Melosky B., Blais N., Cheema P., Couture C., Juergens R., Kamel-Reid S., Tsao M.-S., Wheatley-Price P., Xu Z., Ionescu D. (2018). Standardizing biomarker testing for Canadian patients with advanced lung cancer. Curr. Oncol..

[14] Planchard D., Popat S., Kerr K., Novello S., Smit E., Faivre-Finn C., Mok T., Reck M., Van Schil P., Hellmann M. (2018). Metastatic non-small cell lung cancer: ESMO Clinical Practice Guidelines for diagnosis, treatment and follow-up. Ann. Oncol..

[15] Takano N., Ariyasu R., Koyama J., Sonoda T., Saiki M., Kawashima Y., Oguri T., Hisakane K., Uchibori K., Nishikawa S. (2019). Improvement in the survival of patients with stage IV non-small-cell lung cancer: Experience in a single institutional 1995-2017. Lung Cancer.

[16] Verhagen G.P., Bootsma V.C., Tjan-Heijnen V.C.G., van der Wilt G.J., Cox A.L., Brouwer M.H.J., Corstens F.H.M., Oyen W.J.G. (2004). FDG-PET in staging lung cancer: How does it change the algorithm?. Lung Cancer.

[17] Quoix E., Westeel V., Zalcman G., Milleron B. (2011). Chemotherapy in elderly patients with advanced non-small cell lung cancer. Lung Cancer.

[18] Shayne M., Culakova E., Poniewierski M.S., Wolff D., Dale D.C., Crawford J., Lyman G.H. (2007). Dose intensity and hematologic toxicity in older cancer patients receiving systemic chemotherapy. Cancer.

[19] Repetto L., Audisio R.A. (2006). Elderly patients have become the leading drug consumers: It’s high time to properly evaluate new drugs within the real targeted population. J. Clin. Oncol..

[20] Fakih M., O’Neil B., Price T.J., Falchook G.S., Desai J., Kuo J., Govindan R., Rasmussen E., Morrow P.K.H., Ngang J. (2019). Phase 1 study evaluating the safety, tolerability, pharmacokinetics (PK), and efficacy of AMG 510, a novel small molecule *KRAS^G12C^* inhibitor, in advanced solid tumors. J. Clin. Oncol..

